# Upon the Nature of the Sausage Poison

**Published:** 1855-02

**Authors:** 


### *Upon the nature of the Sausage Poison.*

(Translated for the Medical Examiner.)

The following data are extracted from a notice in Schmidt's Jahrbuch (78, 4) of Prof. Julius Schlossberger's essay upon the *Sausage Poison.* (Vierordt's Arch. xi. 5.)

"The first notice of poisoning by sausage dates from the year 1735. Most of the cases known up to the opening of the present century are found in the Journals of Autenrieth, Kopp, Hufeland, Henke, Rust, Horn, in the works of J. Kerner, and in scattered dissertations which Schlossberger particularly cites. The instances of *poisoning* by *fish* have been principally referred to abroad, by Christison, Lichtenstädt, Orfila, Chevallier and Duchesne.

Sausage poisoning is found, as is well known, to occur principally in *Swabia;* S. estimates the number of cases of sickness produced by sausages in Würtemberg, within the last 50 years, to be at least 400, and the number of deaths from the same cause at least 150. Isolated cases have also been observed in Baden, Bavaria, Hessia, Dessau, Prussia, Saxony, and two cases, not sufficiently confirmed, in France. The poisonings nearly always occur in the winter and spring months, the greatest number proportionately in the month of April, (two-fifths of the whole,) that being the period where those that are eaten have been kept the longest; while in the warm weather, they pass so rapidly into actual putrefaction with the development of stinking gases, as to cause them to be rejected by persons with even the most blunted senses.

*Blood* and *liver* sausages are the only kinds in which the poison ever forms, which S. explains by the mode in which the country people are accustomed to prepare and preserve them. They are, for instance, principally prepared from the blood, brain, liver and similar parts, that are more liable than others to pass quickly into decomposition; are mixed with materials also that easily ferment, such as milk and wheat flour, while the boiling is often incomplete, or too long delayed in warm weather. The smoking also is often incomplete, either from defective smoke houses or from the diameter of the mass being too great, (in the so-called "blungen," or hog's stomach.) In such preparations the stinking putrefaction may be kept off or delayed, but full play is permitted to another species of decomposition the more dangerous, because impossible to be detected by the senses. Besides, these badly smoked sausages are often kept tightly packed in close, confined places. This kind of sausage, moreover, is often prepared by unskilful country butchers, or by the peasants themselves, who, to prevent the intestines from bursting, often purposely commit great errors in boiling and in filling them; too often leave hollows that are filled with fluid, or suffer the sausage to be too greatly penetrated by the broth. All the kinds of blood and liver sausages are of considerably greater diameter than those made from meat, and consequently are more difficult to stuff tightly and to smoke thoroughly (especially in the central postions) than the latter; the poisonous decomposition proceeding evidently from the centre outwards. In place of the blood of swine, that of cattle and of goats is often in part substituted, together with the plucks of sheep. That poisonous properties should likewise be found in such sausages cannot surprise us, when we bear in mind the similarity in composition of the blood and tissues of all the higher animals. Neither spices nor salt, which are generally used in great amount by the country people, prevent the commencement of this species of putrefaction. The character of the intestine used (those of swine and cattle) can have no influence upon the development of the poison, beyond that which arises from imperfect cleansing, and from the too great diameter, rendering the stuffing and drying more difficult.

But few *perceptible physical changes* are exhibited in the poisonous sausage. They often contain in the interior spots like curds or soft cheese, and occasionally are of a crumbly, almost brittle consistence, that extends more and more towards the circumference. Frequently nothing peculiar is presented to the taste or smell; the odor is somewhat disagreeable, resembling that of rancid fat. It is very probable that this odor is due to the formation of volatile fatty acids, since the neutral fats are always present in these sausages, under circumstances favorable to the decomposition of their fatty basis, the protein bodies present serving as ferments, or perhaps giving rise, by their own spontaneous decomposition, to these acids. The taste is described sometimes as sour, sometimes as bitter, or as rancid. A sausage examined by S. showed in its centre softened, curdy places; possessed an acid reaction, (free lactic acid,) an odor resembling butyric or metacetonic acid, and developed, by the addition of weak potash, an ammoniacal and also an extremely disagreeable odor; hydrochloric acid produced therewith copious fumes. The intestine was mouldy; the peripherical layers of the sausage were of normal appearance and not of acid reaction. It is not, however, proved that these softened places are the seat of the poison, many poisonous sausages not possessing them, and it is decided that different portions of the same sausage proved injurious with one individual and harmless with others; and of various spoiled sausages, subjected to the same conditions, some produced symptoms of poisoning, whilst others did not.

The action of the sausage poison upon the human body has, however, been more thoroughly investigated than any other of its properties. The phenomena which it calls forth, consist of functional disturbance of the intestinal canal, of the nervons system, respiratory apparatus, and diminution of the secretions. These symptoms have often been compared with those produced by poisoning with digitalis and with lead. S. considers them of interest, so far as they are capable of throwing light upon the nature of the poison, and directs attention especially to the facts, that the rate of mortality is extremely great; that the poison operates far more fatally upon weak, old, decrepid persons, than upon those otherwise strong and in health; that it is extremely dangerous, even in very small doses, (one or two thin slices of sausage); and that acid fluids appear to heighten the poisonous phenomena.

No constant alterations are found upon the bodies of those that have died from this poison, other than inflammatory spots in the intestinal canal, great muscular rigidity and remarkably slight symptoms of decomposition. In cases where, on the contrary, even during life, evidences of dissolution of the blood and afterwards of rapid decomposition in the corpse are found, it is always due, in part at least, to the use of sausages in stinking putrefaction, and these cases evidently belong to an entirely different class of poisonings.

Nothing satisfactory is as yet known with regard to the action of the sausage poison upon *animals*. S. found that a famishing terrier, which had greedily devoured several ounces of a sausage, (with smeary, softened places,) showed no ill effects from it, after the lapse even of four weeks, although it had fatally sickened a number of men who had eaten of it. This coincides with the experience of many other authors. S. here remarks upon the great necessity of caution in the transference of the deductions from toxicological experiments upon animals to man, and vice versa, particularly with the carnivora, whose intestines, frequently containing large quantities of putrid substances, carrion, &c., require that the gastric juice should possess the most energetic digestive powers. Although Kerner poisoned a large number of animals with substances prepared from spoiled sausages, yet, S. remarks, that those experiments can have in this respect no value, because they were made in part with products from the destructive distillation of hog's fat, and the remainder with impure fatty acids, obtained from the common neutral fats by saponification and subsequent decomposition by the mineral acids, and were in no instance derived from spoiled fats.

According to Buchner and Schumann, who operated with the portions of spoiled sausage, soluble in alcohol, the diluted poison exerts upon animals little or no action; and even when concentrated has much less effect than upon man.

The views that have been formed at various times with regard to the *nature* of this poison, have proved for the most part untenable. For instance, that it is due to an impregnation with metallic poisons; that it is owing to a peculiar decomposition of the sausage forming prussic acid; that it depends upon certain constituents of the smoke, (creasote, pyroligneous acid, &c.;) to the use of poisonous seeds (cocculus indicus) in place of the ordinary species; and finally, that it is due to the production of Welter's bitter (carbazotic acid) or some similar substance. The opinion that the materials used in the noxious sausages have been obtained from diseased animals, is satisfactorily shown in most cases to be without foundation. S. Kerner's views approach nearer the truth, in considering the poison to be produced by a decomposition of the substance of the sausage, not, however, identical with the common forms of putrefaction. But as K. obstinately maintains the fatty nature of the poison, he confounds together the most varied products of the decomposition of the protein bodies with those of the neutral fats. Besides, the margaric and stearic acids, the oily pyrogenous acids and the less volatile fatty acids are not poisonous; at most, the latter are corrosive only in their very concentrated state. Of course, experiments with the products of destructive distillation can lead to no conclusions with regard to the nature of the products of fermentation and putrefaction.

The labors of Buchner and Schumann, who were the first to prepare the aqueous and alcoholic extracts of the sausage, only prove, 1st, that the poison is soluble in alcohol. 2dly, that the alcoholic extract is accompanied with much fat. Liebig represents the sausage poison as being a ferment arising from the putrefaction, and which introduces into the fluids and tissues of the living body its peculiar decomposition, and supports his view by fruitless attempts to isolate the poison, and by the destruction of the poison by treatment with alcohol or boiling water. While, however, Buchner's and Schumann's results rather contradict than confirm Liebig's position, experience has further shown that boiled and baked sausages can cause poisoning, and the phenomena produced (diminution of secretions, &c.) are directly opposite to those produced by the poisons of putrefaction. Since all the theories of the nature of the poison, capable of experimental proof, are not yet exhausted, S. thinks it best to discard the idea of a ferment, as its nature prevents all further investigation, and in place thereof, he proposes his supposition as applicable to most of the cases, and which has already been strengthened by many facts. This theory attributes the action of many poisonous sausages to the presence of an organic base, somewhat similar to nicotine, and is founded upon, 1st, the premises already, in great part, established, that in poisonous sausages and cheese, organic bases are formed by the decomposition of the protein bodies; and 2dly, upon the previous supposition that they give rise to these peculiar symptoms of poisoning, a thought that seems already to have occurred to Kastner, as he suggests the existence of an alkaloid derived from the mould in the sausages. The presence of such volatile bases in the decomposition of nitrogenous animal substances, from which ammonia is subsequently formed, is certainly more than probable, and in many cases stated by Stenhouse, shown to be constant. S. has also found ammonia in large amount in the noxious sausages, and remarked at the same time, a peculiar, disagreeable odor. The behaviour of the greater part of the substances homologous with ammonia, in the organism, is still unknown, and at all events each of them requires a physiological investigation; for out of the innumerable bases that have been and will yet be discovered, many, that are very similar in composition, exert very different effects upon the body. On the other hand, nicotine, coneine and spartein (the three best known representatives of the volatile bases from the vegetable kingdom, and whose close relation with ammonia cannot be ignored) are well known for their extraordinarily poisonous properties. It is certain that alkaloids, like leucine and tyrosine, are found in old cheese, and if these harmless substances occur, why should they not, under certain circumstances, be accompanied by poisonous bodies possessing the same chemical character? S. here observes, that opium, together with bases that act powerfully upon the organism, also contains alkaloids that are perfectly indifferent in this respect. S. also cites the instance of the volatile bases that Wertheim and Hofmann have shown to exist in herring pickle (propylamine, trimethylamine). S. finally endeavors to make use, for his hypothesis, of the circumstance discovered by Kerner and A——, that the products of destructive distillation of fresh blood-sausages cause similar symptoms in animals to those of the sausage poisoning. He there points out (according to Anderson's investigations upon the pyrogenous oil from bones) that during the destructive distillation of nitrogenous bodies, together with the so-called empyreumatic bases, the alcohol bases, such as methyl, æthylamine, &c., also appear, and that these, (like the separation of leucine into valerianic acid and ammonia,) form the necessary steps, in place of the fatty acids and ammonia. S. would also seek for the same volatile bases in poisonous mushrooms, in ergot, in rotten potatoes, in the air of graves and cloacæ, and even in the so-called cadaveric poison, and considers them as the cause of the action of these substances upon the organism.—*Not. fur prakt. Arzte.* 1854. *Band* 6. *S.* 485.

